# Supplementary material for: Human Blood Serum Can Diminish EGFR-Targeted Inhibition of Squamous Carcinoma Cell Growth through Reactivation of MAPK and EGFR Pathways
Source: Cells. 2023 Aug 8;12(16):2022. doi: 10.3390/cells12162022 (PMC10453612; doi:10.3390/cells12162022)
Supplement: Supplementary file 1 [file cells-12-02022-s001.zip › Supplemental Data.pdf]

**Human blood serum can diminish EGFR-targeted inhibition of squamous carcinoma cell growth through reactivation of MAPK and EGFR pathways**

Dmitry Kamashev, Nina Shaban, Timofey Lebedev, Vladimir Prassolov, Maria Suntsova, Mikhail Raevskiy, Nurshat Gaifullin, Marina Sekacheva, Andrew Garazha, Elena Poddubskaya, Maksim Sorokin, Anton Buzdin.

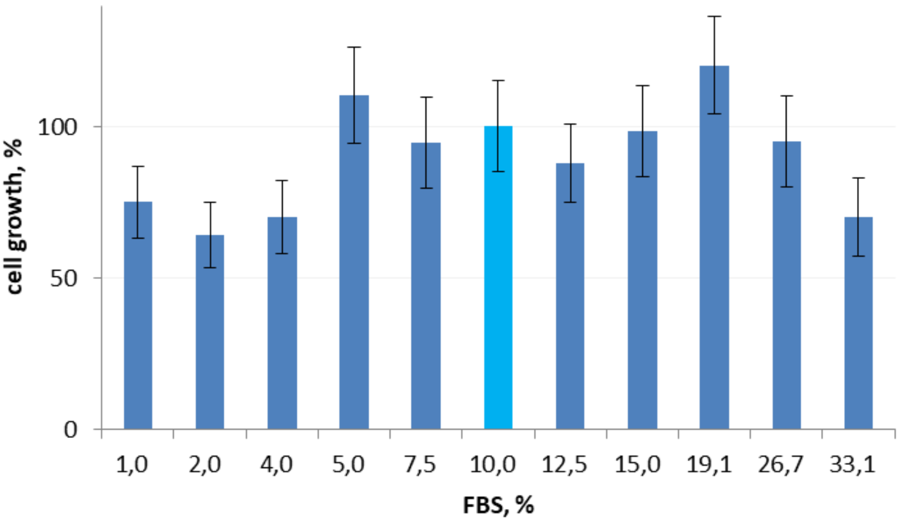


Figure S1 A431 cell growth during 4-day treatment with the DMEM supplemented with indicated (in %) amount of FBS. Bars represent average cell count for each FBS concentration calculated from three replicates normalized to 10% FBS.

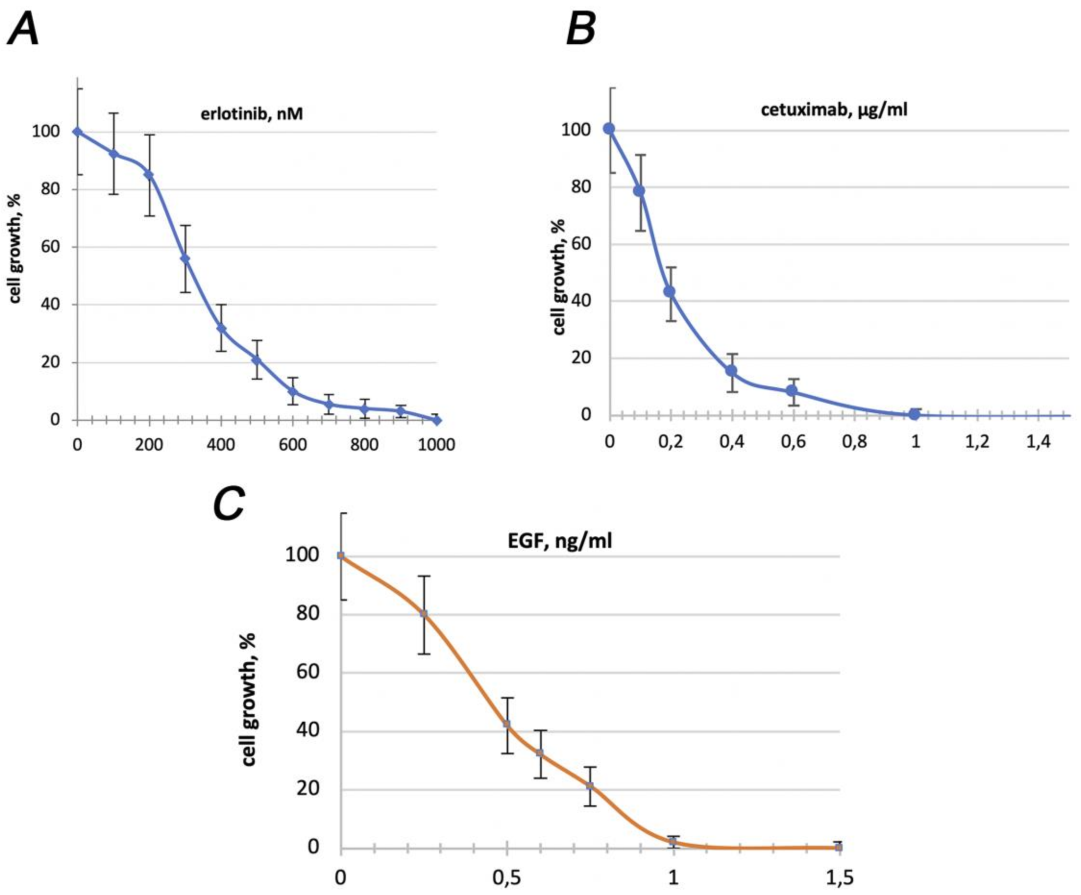


Figure S2. A431 cell growth during 4-day treatment with the medium supplemented FBS (10%) and: Panel A, erlotinib (in nM); Panel B, cetuximab (in µg/ml), Panel C, EGF (in ng/ml). Curves show average of cell count calculated from three replicates normalized to non-drug conditions.

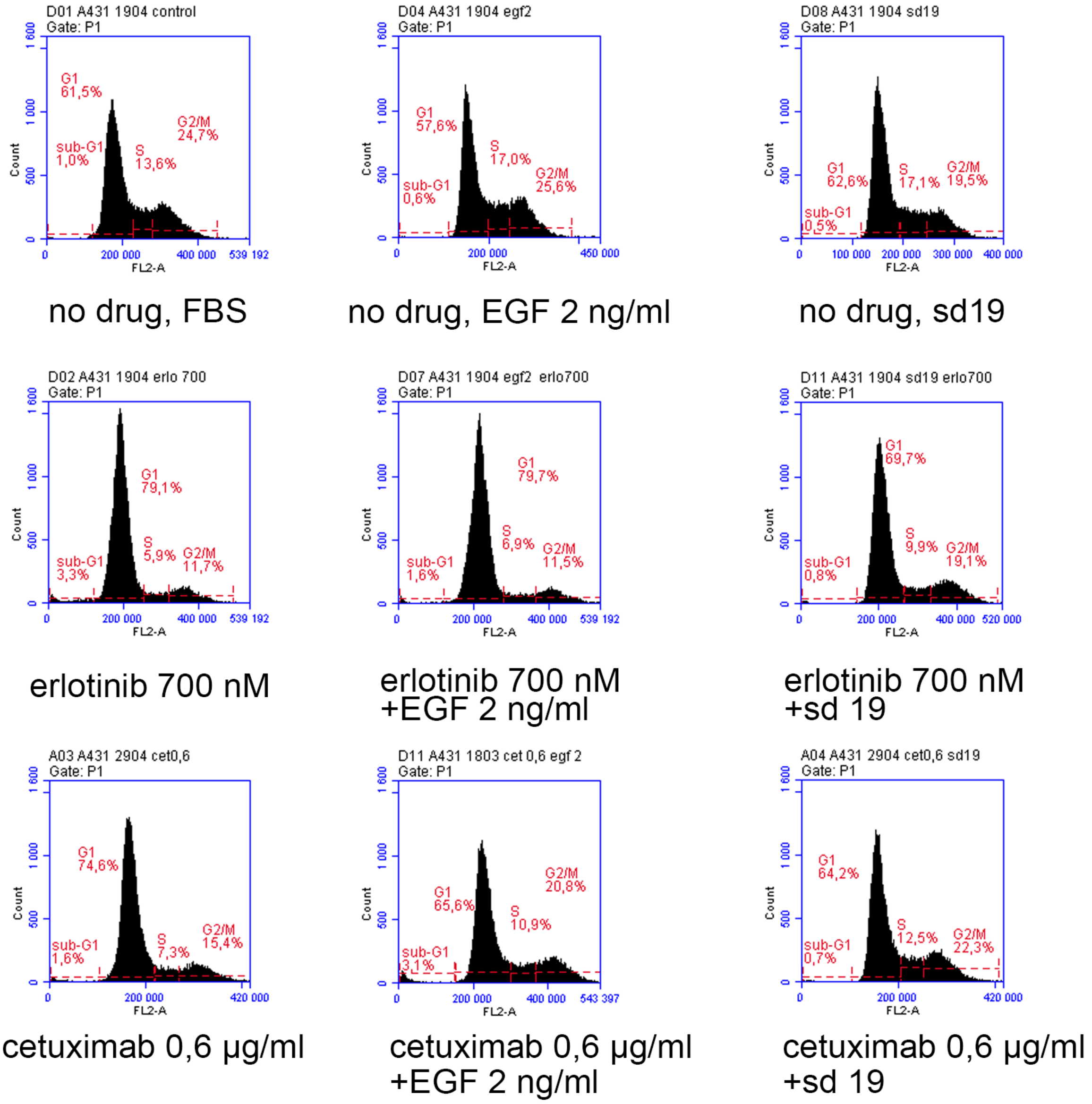


Figure S3. The effect of erlotinib, cetuximab, EGF or 5 % human blood serum (sd19) on cell cycle distribution in A431 cells. Cells were treated with the indicated concentrations of targeted drugs for 72 h, stained with PI (propidium iodide), and then subjected to flow cytometric analysis to determine their distribution in each phase of the cell cycle. Historgrams represent DNA content (x axis, PI fluorescence) versus counts (y axis).

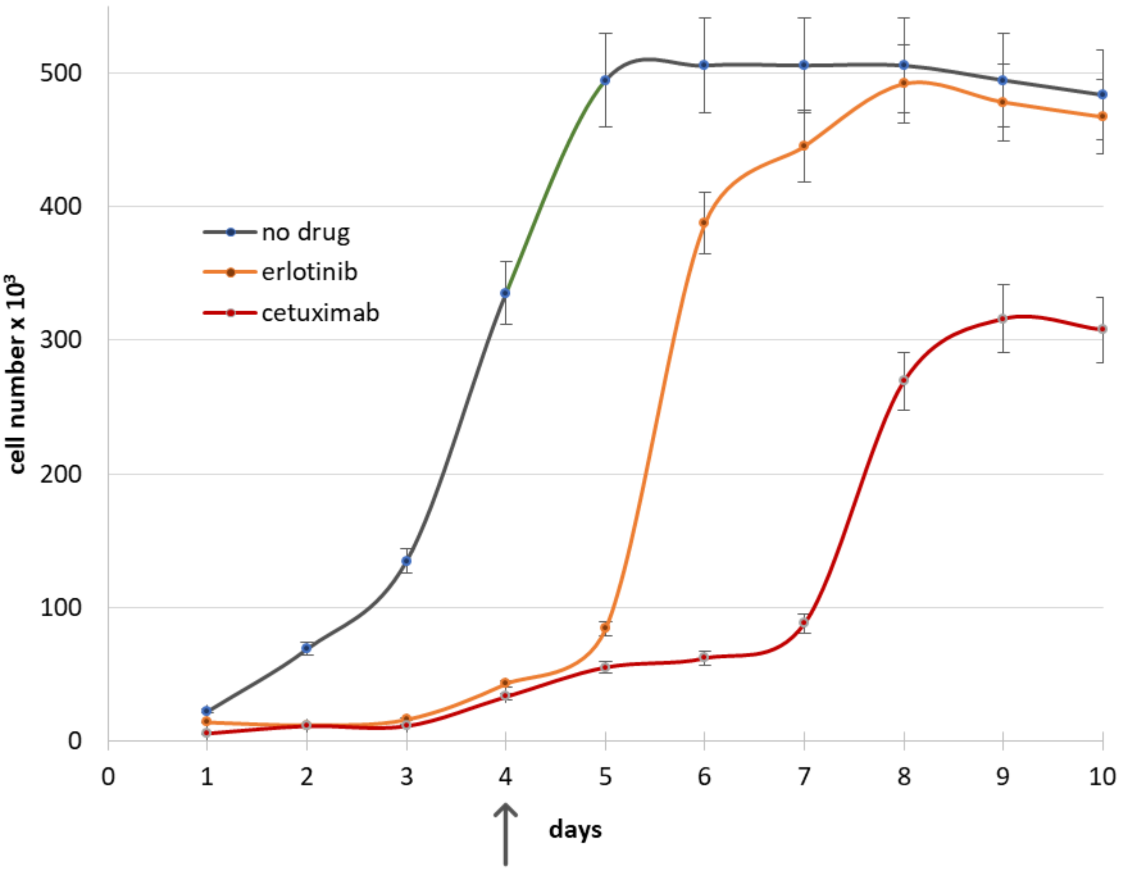


Figure S4. A431 growth rate after treatment with cetuximab (0.82 µg/ml) and erlotinib (700 nM). On the day 4 (indicated by the arrow) the drug was removed and cells were further incubated in the drug-free media. Curves represent average cell number for every day, the numbers were calculated from three replicates.

Cetuximab and erlotinib treatment inhibits A431 cell growth in reversible manner.

To choose experimental conditions for cell treatment for gene expression profiling, we measured cell growth rate following to drug removal to be sure that three-day drug treatment does not change cell fate irreversibly. We chose the cetuximab concentration of 0.82 µg/ml and erlotinib concentration of 700 nM. Under these conditions both drugs completely arrest the cell growth. Cells were treated for three days, and medium was changed to the fresh drug-free medium. Growth rate curves show that after drug removal cells still do not proliferate for four more days after erlotinib treatment and for seven days after cetuximab treatment; and then cells boost to growth with the rate close to the rate for non-treated cells; cells reach confluence with the density ∼ 60% (cetuximab treatment) or with density ~95% (erlotinib treatment) compared with non-treated cells. These results ensure that two- or three-day treatments with both drugs remain cells intact and that the drug treatment is reversible.

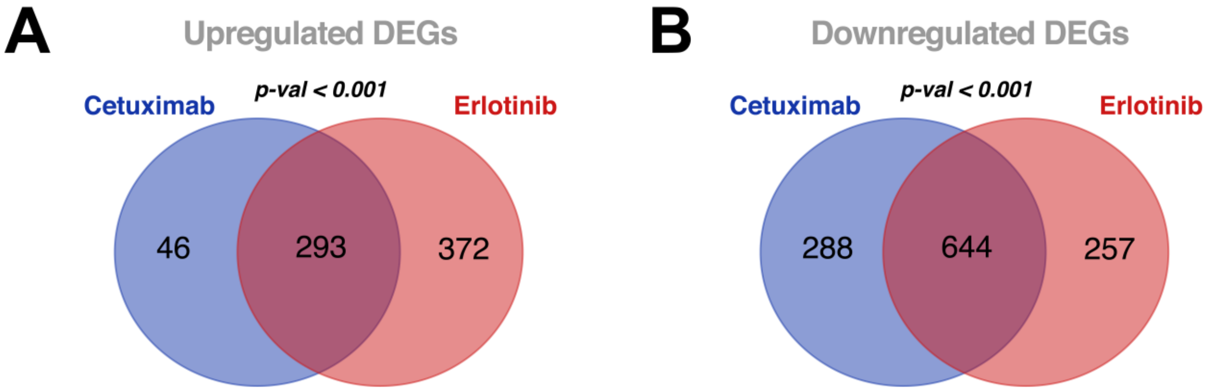


Figure S5. Venn diagrams represent the overlap of upregulated (Panel A) and downregulated (Panel B) genes (DEGs) in the presence of cetuximab only and erlotinib only.

Table S1. Alteration of cell-cycle distribution upon the treatment with erlotinib, cetuximab, EGF and human blood serum (5%) of A431 cells compared with non-treated cells, in % #.

| Treatment | ΔG0/G1 | signi ficance | ΔS | signi ficance | ΔG2/M | signi ficance |
|---|---|---|---|---|---|---|
| EGF 2 ng/ml | -0,493 | | -0,949 | | 1,443 | |
| EGF 8 ng/ml | 7,206 | *** | -3,214 | | -3,993 | * |
| sd19 | 2,766 | | 1,639 | | -4,406 | ** |
| sm1 | 0,378 | | 1,141 | | -1,519 | ** |
| sd14b | 0,583 | | 1,348 | | -1,931 | * |
| sd15 | -1,421 | | 1,17 | | 0,251 | |
| mean sd | 0,577 | | 1,324 | *** | -1,901 | |
| erlotinib 700 nM with: | 19,761 | *** | -7,907 | *** | -11,854 | *** |
| EGF 2 ng/ml | 19,729 | *** | -5,313 | * | -14,416 | *** |
| EGF 8 ng/ml | 20,694 | *** | -6,784 | *** | -13,91 | *** |
| sd19 | 8,259 | *** | -4,087 | ** | -4,172 | *** |
| sm1 | 7,049 | *** | -3,076 | | -3,973 | *** |
| sd14b | 12,396 | ** | -5,543 | | -6,854 | *** |
| sd15 | 12,117 | *** | -5,662 | *** | -6,455 | *** |
| mean sd+ erlotinib | 9,955 | *** | -4,592 | *** | -5,363 | *** |
| cetuximab 0,6 µg/ml with: | 14,876 | *** | -8,184 | *** | -6,691 | *** |
| EGF 2 ng/ml | 5,445 | *** | -2,384 | *** | -3,06 | *** |
| EGF 8 ng/ml | -1,781 | | 0,566 | | 1,215 | |
| sd19 | 5,145 | | -0,478 | | -4,667 | |
| sm1 | 2,658 | | -2,702 | *** | 0,045 | |
| sd14b | 9,014 | *** | -3,078 | ** | -5,936 | *** |
| sd15 | 5,889 | * | -3,87 | *** | -2,019 | |
| mean cet+sd | 5,676 | * | -2,532 | | -3,144 | |

# In the non-treated A431 cells, the proportion of G0/G1 cells was 61.58 ± 2.15; in the S phase, the proportion was 13.91 ± 2.41; in the G2/M phase, the proportion was 24.44 ± 11.68 (%). Comparisons of treated and non-treated cells are indicated as: *,p<0,05; **,p<0,01; ***,p<0,001

Table legends:

**Table S2.** Comparison of gene expression between A431 cells treated with cetuximab + human blood serum (sd); cetuximab + EGF; cetuximab only, EGF; and human blood (sd), and non-treated cells (no) revealed by RNA-Seq analysis columns 2 - 8. Comparison of gene expression between A431 cells treated with erlotinib + human blood serum (sd); erlotinib + EGF; erlotinib only, EGF; and human blood (sd), and non-treated cells (no) revealed by RNA-Seq analysis columns 9 – 15. Comparison of gene expression between A431 cells treated with human blood (sd) and non-treated cells (no), column 16; and with EGF and non-treated cells (no), column 17.

The table lists the Genes symbols (first column) and a log2 (fold change) for the genes with fold change of least 2 and p-value < 0.05. Genes which belongs to Cetuximab core group and Erlotinib core group are marked in columns 19 and 20.

**Table S3.** Pathway activation level (PAL) calculated on the basis of RNA sequencing of treated A431 cells. PALs were calculated to compare pairs of all possible treatment conditions. Only pathways not essentially affected by the presence of EGF or human serum along with EGFR-targeted drugs are shown. Pathways not significantly altered between compared treatments are indicated as NA, human blood serum is indicated as sd.

**Table S4.** DEGs (cetuximab vs no-drug (no)), which are not differentially expressed in the presence of EGF or human serum (cetuximab+EGF vs no and cetuximab + human blood serum vs no, erlotinib+EGF vs no and erlotinib + human blood serum), totally 127 genes, revealed by RNA-Seq analysis. The table lists the DEGs, i.e. genes with a fold change (FC) of at least 2 and p-value < 0.05.  The table lists the genes and a log2 (fold change).  Gene symbols are indicated in columns 1. Gene annotations are taken from www.genecards.org/, www.uniprot.org, www.proteinatlas.org, www.omim.org/ (columns 21-26).
